# Polycyclic aromatic hydrocarbon components contribute to the mitochondria-antiapoptotic effect of fine particulate matter on human bronchial epithelial cells *via *the aryl hydrocarbon receptor

**DOI:** 10.1186/1743-8977-7-18

**Published:** 2010-07-21

**Authors:** Ioana Ferecatu, Marie-Caroline Borot, Camille Bossard, Melanie Leroux, Nicole Boggetto, Francelyne Marano, Armelle Baeza-Squiban, Karine Andreau

**Affiliations:** 1 Université Paris Diderot-Paris 7. Unit of Functional and Adaptive Biology (BFA) CNRS EAC 4413, Laboratory of Molecular and Cellular Responses to Xenobiotics, Bâtiment Buffon, case courrier 7073, 5 rue Thomas Mann, 75013 Paris, France; 2 Université Paris Diderot-Paris 7. Flow Cytometry Unit, Jacques Monod Institute, 75013 Paris, France

## Abstract

**Background:**

Nowadays, effects of fine particulate matter (PM_2.5_) are well-documented and related to oxidative stress and pro-inflammatory response. Nevertheless, epidemiological studies show that PM_2.5 _exposure is correlated with an increase of pulmonary cancers and the remodeling of the airway epithelium involving the regulation of cell death processes. Here, we investigated the components of Parisian PM_2.5 _involved in either the induction or the inhibition of cell death quantified by different parameters of apoptosis and delineated the mechanism underlying this effect.

**Results:**

In this study, we showed that low levels of Parisian PM_2.5 _are not cytotoxic for three different cell lines and primary cultures of human bronchial epithelial cells. Conversely, a 4 hour-pretreatment with PM_2.5 _prevent mitochondria-driven apoptosis triggered by broad spectrum inducers (A23187, staurosporine and oligomycin) by reducing the mitochondrial transmembrane potential loss, the subsequent ROS production, phosphatidylserine externalization, plasma membrane permeabilization and typical morphological outcomes (cell size decrease, massive chromatin and nuclear condensation, formation of apoptotic bodies). The use of recombinant EGF and specific inhibitor led us to rule out the involvement of the classical EGFR signaling pathway as well as the proinflammatory cytokines secretion. Experiments performed with different compounds of PM_2.5 _suggest that endotoxins as well as carbon black do not participate to the antiapoptotic effect of PM_2.5_. Instead, the water-soluble fraction, washed particles and organic compounds such as polycyclic aromatic hydrocarbons (PAH) could mimic this antiapoptotic activity. Finally, the activation or silencing of the aryl hydrocarbon receptor (AhR) showed that it is involved into the molecular mechanism of the antiapoptotic effect of PM_2.5 _at the mitochondrial checkpoint of apoptosis.

**Conclusions:**

The PM_2.5_-antiapoptotic effect in addition to the well-documented inflammatory response might explain the maintenance of a prolonged inflammation state induced after pollution exposure and might delay repair processes of injured tissues.

## Background

Nowadays, air pollution is considered as a major inducer of harmful health effects, especially due to fine particulate matter (PM_2.5_, atmospheric particles with an aerodynamic diameter equal or less than 2.5 μm). Urban PM_2.5 _is a mixture composed mainly of soots from fossil fuel combustion [1] together with several components adsorbed, including organic elements, biological species and metals [2]. *In vitro *short-term exposure to PM is associated with an inflammatory response as a consequence of cellular oxidative stress increase [3]. Fine PM are taken up by airway epithelial cells and alveolar macrophages [4,5] leading to proinflammatory cytokine expression and release (i.e. GM-CSF, IL-1α, IL-8, TNFα, etc) [6,7] as well as the production of reactive oxygen species (ROS) [8]. Moreover, recent data demonstrate that short exposure of bronchial or nasal epithelial cells to urban PM_2.5 _provokes the secretion of EGFR ligands and Amphiregulin, which leads to GM-CSF secretion *via *an autocrine pathway [9].

Long-term effect of atmospheric particles remains underestimated. Nevertheless, epidemiological studies provide evidence of their deleterious impacts by increasing cardiopulmonary morbidity and mortality [10], asthma [11], bronchitis [12], exacerbation of chronic obstructive pulmonary disease (COPD, [13]). In addition, cancerous pathologies such as tracheal, bronchial and lung tumors are exacerbated [14]. In tissues, chronic exposure was associated with persistence of particles into the lungs leading to bronchioli wall thickening [15] and airway remodeling characterized by epithelial mucus-producing cells metaplasia, subepithelial fibrosis and airway smooth muscle hypertrophy/hyperplasia as observed in chronic asthma and COPD [16]. Thus, mechanisms involved in airway remodelling might be the excessive cell proliferation as well as the resistance to the apoptotic cell death.

Apoptosis is a programmed cell death defined by specific morphological alterations but with only slight ultrastructure modifications of cytoplasmic organelles and phosphatidylserine (PS) residue externalization [17]. It is noteworthy that mitochondrial alterations constitute the checkpoint of the apoptotic cell death. This is highlighted by the mitochondrial membrane permeabilization (MMP) which is measured by the decrease of mitochondrial transmembrane potential (ΔΨm), and by the subsequent superoxide anion production and Cytochrome *c *release. The activation of caspases or other proteases triggers the proteolysis of specific substrates involved into the final appearance of morphological features of apoptosis. Most publications dealing with toxicity of airborne particles showed an induction of apoptosis associated with ROS generation, ΔΨm drop, caspase-9 activation and DNA fragmentation [18]. *In vitro *experiments showed that PM-induced apoptosis was reported in normal human lung tissue or airway epithelial cells [19,20].

The toxicity of ambient particles is mainly attributed to various adsorbed components. For instance, organic compounds are known to mimic the apoptotic effect of PM in various cell types through pathways which require the activation of the aryl hydrocarbon receptor (AhR) and the generation of ROS leading to DNA damage. Nevertheless, polycyclic aromatic hydrocarbon (PAH) induced-apoptosis is mainly mediated *via *the mitochondria pathway in a p53-dependent manner [21]. Metals also affect human health, especially when these toxicants compete with essential elements and modify many cellular processes. Transition metals promote apoptosis through ROS generation, mitochondria dysfunction, activation of MAPK, p53 and caspases or down regulation of antiapoptotic proteins Bcl-2 [22]. Metals and the water-soluble fractions of PM are known to cause inflammation and cancer mostly due to DNA damage as a consequence of ROS generation by Fenton reaction. In addition, the exacerbation of asthma after inhalation of PM is mainly attributed to the biological compounds. Endotoxins induce proinflammatory cytokines production [23] and are able to provoke apoptosis-like cell death involving a scavenger receptor.

Most of PM pro-apoptotic data were obtained *in vitro *from acute exposure (with 80 to 100 μg/cm^2 ^of particles) which usually corresponds to high pollution periods. The purpose of the present study was to investigate the effect of low doses of air particles (PM_2.5_), on different bronchial epithelial cells (tumoral, immortalized and primary cells) regarding their induction or reduction of apoptosis. First, we found that Parisian PM_2.5 _are not cytotoxic, but have an antiapoptotic effect towards well-known cell death inducers, A23187, staurosporine and oligomycin. The reduced apoptosis observed after particle exposure is not related to the pro-inflammatory response and the EGF pathway. Moreover, water-soluble as well as organic components such as heavy PAH, are able to mimic the effects triggered by PM_2.5_, suggesting that such compounds are involved in the antiapoptotic effect. Finally, we identified the aryl hydrocarbon receptor as a molecular effector involved in the mechanism of the antiapoptotic effect of PM_2.5 _on human bronchial epithelial cells.

## Results

### PM_2.5 _are not cyctotoxic in human bronchial epithelial cells

**Figure 1:**
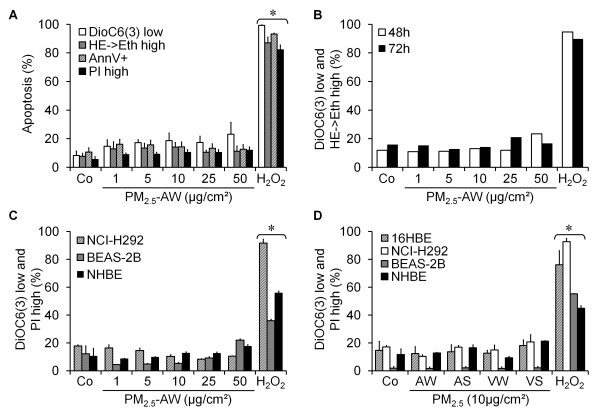
**Effect of PM_2.5_-AW exposure on human bronchial epithelial cells**. (A) Dose response of DiOC6(3) (to measure the mitochondrial ΔΨm drop), hydroethidine (HE, ROS-sensitive dye), Annexin V-FITC (for phosphatidylserine exposure) and the propidium iodide (PI, as a plasma membrane permeabilization marker) staining after 24 hours exposure of 16HBE bronchial epithelial cells to PM_2.5_-AW (1-50 μg/cm^2^) or H_2_O_2 _(500 μM). (B) Kinetic study of PM_2.5_-AW effect on apoptosis. 16HBE cells were exposed to PM_2.5_-AW (1 to 50 μg/cm^2^) or H_2_O_2 _(500 μM) for 48 h or 72 h before flow cytometric analysis of cells presenting simultaneously a mitochondrial depolarization (DiOC6(3) low) and a superoxide anion generation (HE -> Eth high). Results are representative of three independent experiments. (C) Subconfluent bronchial epithelial cell lines NCI-H292, BEAS-2B and primary bronchial epithelial cells (NHBE) were exposed 24 h to 1-50 μg/cm^2 ^PM_2.5_-AW, and H_2_O_2 _(1 mM) before flow cytometric analysis of cells presenting simultaneously a DiOC6(3) low and PI high staining. (D) Human bronchial epithelial cells 16HBE, NCI-H292, BEAS-2B or NHBE were exposed 24 hours to 10 μg/cm^2 ^of different batches of PM_2.5 _(Auteuil-Winter (AW), Auteuil-Summer (AS), Vitry-Winter (VW) or Vitry-Summer (VS) corresponding to two locations of Paris: (i) a school playground at Vitry-sur-Seine in a suburb of Paris and (ii) Porte d'Auteuil adjacent to a major highway). Then, cells were analyzed as previously described. Data are represented as mean ± SD (* treated vs. control, *p *< 0.05, n = 3).

First, we were interested in finding out whether particles from Parisian ambient air have cytotoxic effect on human bronchial cells. Thus, we exposed 16HBE human bronchial epithelial cells to increasing amount of PM_2.5_-AW from 1 to 50 μg/cm^2^. Several hallmarks of apoptotic cell death - recommended by the Nomenclature Committee on Cell Death [17] - were quantified by flow cytometry. Figure 1A shows that 24 h exposure to PM_2.5_-AW induced none of several hallmarks of apoptosis such as: ΔΨm drop (as quantified with DiOC6(3) low staining), oxidative potential (as quantified with the superoxide anion-reactive hydroethidine), phosphatidylserine exposure (driven by Annexin V-FITC positive staining) and plasma membrane permeabilization (allowing propidium iodide high staining). H_2_O_2 _is used here as positive control of apoptosis. Moreover, even when 16HBE cells were exposed for longer times to PM_2.5_-AW (1-50 μg/cm^2^), no significative increase of apoptotic parameters was observed (Figure 1B) suggesting that PM_2.5_-AW do not have cytotoxic activity on human bronchial epithelial cells 16HBE exposed for 24 up to 72 hours. In order to determine if this lack of toxicity is specific to 16HBE cells, we extended our study to other human bronchial epithelial cells, such as NCI-H292 and BEAS-2B cell lines and to non-differentiated primary human bronchial epithelial cells (NHBE). Similarly to 16HBE cells, the dose effect study (1-50 μg/cm^2^) of PM_2.5_-AW did not show any induction of apoptotic cell death, measured by ΔΨm loss and PI high staining, with any of the three different cell types tested (Figure 1C). Conversely, cells tested herein were not resistant to apoptosis induction as demonstrated after 24 h incubation with hydrogen peroxide.

These results might be related to the batch of PM_2.5 _used, in particular timing and location of particle collection. To test this hypothesis, we used several batches of Parisian PM_2.5_: Auteuil-Winter (AW), Auteuil-Summer (AS), Vitry-Winter (VW) or Vitry-Summer (VS) collected in the Paris area: (i) Porte d'Auteuil adjacent to a major highway and considered as a curbside station and (ii) a school playground at Vitry-sur-Seine in the suburb of Paris. When bronchial cells were exposed 24 h to these PM_2.5 _(10 μg/cm^2^), we noticed only an increased granularity corresponding to particle uptake without any reduction in cell size (data not shown). Apoptotic cell death was then quantified by ΔΨm loss and plasma membrane permeabilization, and none of these parameters was significantly increased by exposure to the four different batches of PM_2.5 _(Figure 1D). Altogether, Parisian PM_2.5 _seem to have no cytotoxic effect in several human bronchial epithelial cells, including the primary NHBE cells.

### Parisian PM_2.5 _have an antiapoptotic effect

**Figure 2:**
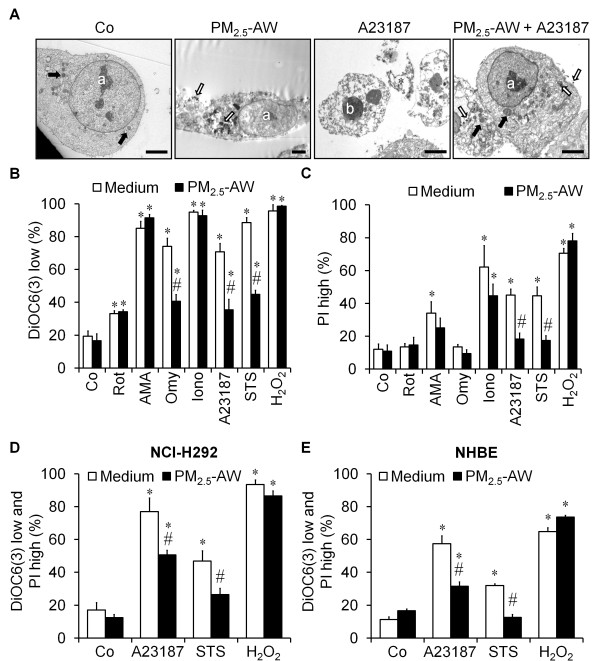
**Parisian PM_2.5 _have an antiapoptotic effect against several death inducers**. (A) Transmission electron microscopy of 16HBE cells treated 24 h with A23187 (3 μM) in the absence or in the presence of a 4 h pretreatment with 10 μg/cm^2 ^PM_2.5_-AW. The micrographs illustrate that control or PM_2.5_-treated cells show normal nuclear chromatin condensation with the presence of some nucleoli (a) and normal ultrastructure of mitochondria (black arrow). PM's aggregates are localized near to the plasma membrane of cells exposed to particles or into the cytoplasm (white arrows). Treatment with A23187 alone triggered typical features of apoptotic cell death characterized by reduced cellular volume, massive chromatin condensation (b), formation of apoptotic bodies (black asterisk) and maintenance of the plasma membrane integrity. Note that PM_2.5_-AW pretreatment prevented A23187-induced morphological modifications and allowed 16HBE cells to retain nuclear or mitochondria morphologies similar to those of the control. Scale bar represent 5 μm. (B and C) After a 4 h pretreatment with PM_2.5_-AW (10 μg/cm^2^), 16HBE cells were exposed to different inducers of cell death for another 20 h such as mitochondrial respiratory chain inhibitors (rotenone (Rot, 5 μM), antimycin A (AMA, 25 μg/ml) and oligomycin (Omy, 5 μM)), calcium ionophores (ionomycin (Iono, 0.5 μM) and A23187, 3 μM), protein kinases inhibitor (staurosporine STS, 1 μM) and oxidative stress activator (H_2_O_2_, 500 μM). Then, cells were quantified for DiOC6(3) low or PI high staining by flow cytometry. (D and E) The human bronchial epithelial NCI-H292 cell line and NHBE primary cells were assessed by flow cytometry in the presence or the absence of PM_2.5_-AW pretreatement (10 μg/cm^2^) and apoptosis inducers: A23187 (3 μM), staurosporine (STS, 1 μM) and H_2_O_2 _(500 μM). Experiments were repeated three times, and means ± S.D. are shown. Significance was calculated with respect to untreated controls (*, *p *< 0.001) and with respect to non-pretreated cells with particles (#, *p *< 0.001).

The lack of cytotoxicity of PM_2.5 _on 16HBE does not mean that atmospheric particles do not modify the state of bronchial cells, for instance the capacity to die by apoptosis. Indeed, some components adsorbed on PM_2.5 _are well-known modulators of the apoptotic process. To determine whether PM_2.5 _were able to reduce cell death, 16HBE cells were exposed 24 h to A23187 (3 μM), a calcium ionophore known to induce apoptosis acting through endoplasmic reticulum and mitochondria stress in HeLa cells [24]. A transmission electron microscopy study of 16HBE cells exposed to A23187 showed typical morphological alterations of apoptosis such as reduction in cellular volume, nuclear chromatin condensation (pyknosis), organelle modifications, but with maintenance of the plasma membrane integrity (Figure 2A). In agreement with previous results, particle exposure alone did not alter 16HBE ultrastructure. However, when PM_2.5_-AW were added 4 h prior to A23187, particles prevented apoptotic alterations and maintained nuclear and mitochondrial morphologies similar to the control condition. Moreover, A23187 alone provoked the reduction of cell size (66.2% of control) and increased granularity (149.2% of control) but PM_2.5_-AW (50 μg/cm^2^) totally counteracted the cellular volume decrease (data not shown).

**Figure 3:**
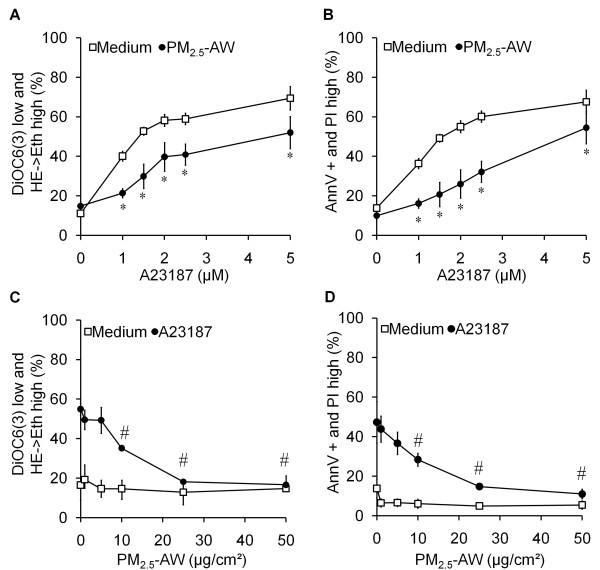
**Dose-effect studies of the antiapoptotic effect of PM_2.5_-AW**. Apoptosis was quantified simultaneously by four parameters and expressed as percentage of 16HBE cells showing ΔΨm drop (DiOC6(3) low) and ROS production (HE->Eth high) or PS exposure (Annexin V+) and plasma membrane permeabilization (PI high). (A and B) After a 4 h pretreatment with PM_2.5_-AW (10 μg/cm^2^), 16HBE cells were exposed to the indicated concentrations of A23187. Significance was calculated with respect to medium conditions (*, *p *< 0.001, n = 3). (C and D) Particle pretreatment was performed with different doses (1-50 μg/cm^2^) 4 h prior to induction of apoptosis by 3 μM of A23187. Results are means ± S.D (n = 3). Significance was calculated with respect to non-pretreated cells with particles (#, *p *< 0.001).

These results strongly suggest that PM_2.5 _might have an antiapoptotic effect. To test this, we used widespread cell death inducers directed against different organelles or effectors of apoptosis such as: three mitochondrial respiratory chain inhibitors (rotenone, antimycin and oligomycin), two calcium ionophores (ionomycin and A23187), a protein kinase inhibitor (STS), and an oxidative stress inducer (hydrogen peroxide, Figure 2B and 2C). A 4 h pretreatment with PM_2.5_-AW allowed a significant reduction of apoptosis induced by the ATP synthase inhibitor oligomycin (33% reduction of DiOC6(3) low), the calcium ionophore A23187 (35% reduction of DiOC6(3) low) and staurosporine (44% reduction of DiOC6(3) low), but not by ionomycin, rotenone, antimycin A and H_2_O_2_. Furthermore, experiments performed in NCI-H292 and NHBE cells showed that PM_2.5_-AW also reduced apoptosis induced by A23187 or STS but not by H_2_O_2 _suggesting that the antiapoptotic effect of atmospheric particles could be a general feature of human bronchial epithelial cells (Figure 2C and 2D). Toxicological studies showed that PM_2.5_-AW significantly prevented mitochondrial and plasma membranes alterations of apoptosis at concentrations as high as 5 μM of A23187 (Figure 3 A and 3B). Moreover, the antiapoptotic effect of PM_2.5_-AW was partially efficient at 10 μg/cm^2 ^and totally effective for concentrations beyond 25 μg/cm^2 ^(Figure 3 C and 3D) suggesting that the antiapoptotic activity of PM_2.5 _is effective at the mitochondrial checkpoint.

**Figure 4:**
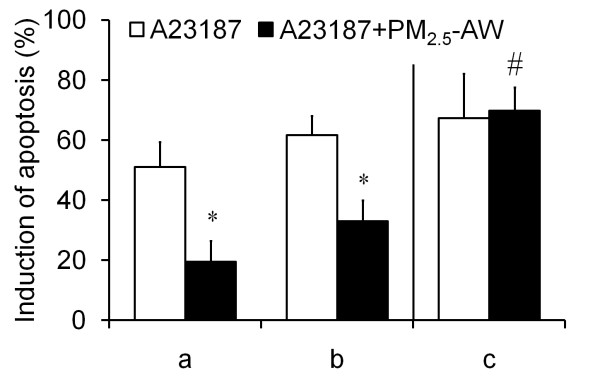
**Study of direct adsorption of A23187 onto particles**. (a) 16HBE cells were exposed 4 h to 10 μg/cm^2 ^PM_2.5_-AW prior to addition of 3 μM A23187 during 20 supplementary hours. (b) To avoid possible adsorption, PM_2.5_-AW were removed and cells were washed five times with PBS before drug treatment. (c) Particles and A23187 were co-incubated 4 h into a coated 24 wells-plate without cells, and then supernatant was used to trigger apoptosis for 20 h supplementary. ΔΨm dissipation was measured by flow cytometry and results are expressed as percentage of apoptosis induction as described in Materials and Methods section. Results are means ± S.D (n = 3). Significance was calculated for PM_2.5_-AW + A23187 versus A23187 alone for all conditions with *p *< 0.05 (*) or for PM_2.5_-AW + A23187 treatment with respect to a, b or c condition (#, *p *< 0.05).

Recently, we showed that nanoparticles are responsible for cytokines adsorption (such as GMC-SF and TNFα) as well as other proteins like fetal calf serum or bovine serum albumin [25]. To investigate if the reduction in A23187-mediated apoptosis observed with PM_2.5 _pretreatment was not due to a possible adsorption of A23187 onto particles, we performed two different experiments. On one hand, inhibition of apoptosis was still maintained when cells were first exposed to PM_2.5 _followed by several washes before addition of the apoptotic inductor (Figure 4a and 4b). On the other hand, PM_2.5 _and the apoptotic inducer were incubated together without cells. After sedimentation, the supernatant containing the nonadsorbed inducer was added to 16HBE cells. This does not seem to reduce the apoptotic effect of A23187 (Figure 4c). Altogether, these two experiments show that the apoptotic resistance is not related to the adsorption onto PM_2.5 _but rather suggest a specific molecular mechanism occurring in bronchial epithelial cells.

### The antiapoptotic effect of PM_2.5 _is related to organic and water-soluble components

**Figure 5:**
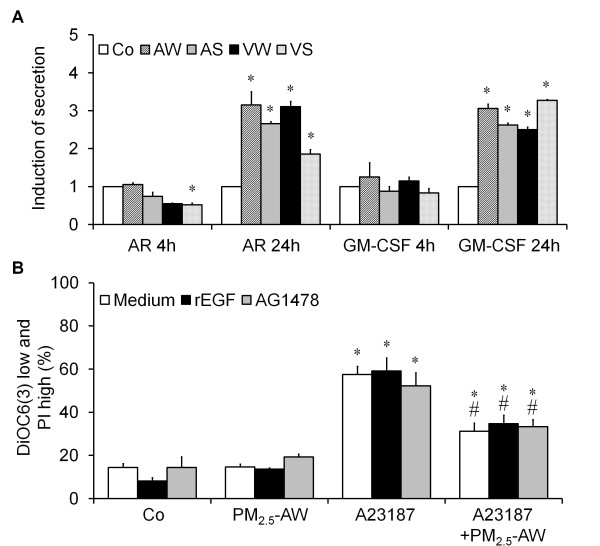
**The antiapoptotic effect is not correlated with proinflammatory cytokines release after PM_2.5 _exposure**. (A) Proinflammatory potential of different batches of PM_2.5 _10 μg/cm^2 ^(AW, AS, VW and VS) on 16HBE cells. After 4 h or 24 h particle exposure, Amphiregulin (AR) and granulocyte monocyte colony-stimulating factor (GM-CSF) secretion were evaluated by ELISA. Data are represented as mean ± SD of triplicates. * indicates significance at *p *< 0.001, treated vs. control. (B) Flow cytometric analysis was done as before in the presence or absence of PM_2.5_-AW pretreatement and/or apoptosis inducer A23187 (3 μM). The implication of the EGFR was evaluated using the recombinant EGF ligand (rEGF 150 ng/ml) or the inhibitor AG1478 (1 μM). Data are represented as mean ± SD of three independent results (* treated vs. control *p *< 0.05; **# **vs. A23187 alone, *p *< 0.05).

Several studies on atmospheric particles underlined that cytotoxic effect of PM were linked to an oxidative stress and secretion of proinflammatory cytokines *via *the epidermal growth factor receptor (EGFR) ligands such as Amphiregulin (AR). Thus, we analyzed the secretion of GM-CSF and AR after performing a 4 h or a 24 h PM_2.5 _exposure (Figure 5A). Results showed that AR and GM-CSF secretion occur only after a 24 h exposure, which is in agreement with previous studies published on PM_2.5_-VW and PM_2.5_-AS [9,26]. Our results suggest that the antiapoptotic activity of PM_2.5_, which is an early event, is not related to the EGFR pathway and secretion of proinflammatory cytokines which is a late event. To confirm this, we used a recombinant EGF ligand (rEGF) or the inhibitor of EGF receptor (AG1478, Figure 5B) to show that none of the two compounds modifies the reduction of A23187-induced apoptosis.

**Figure 6:**
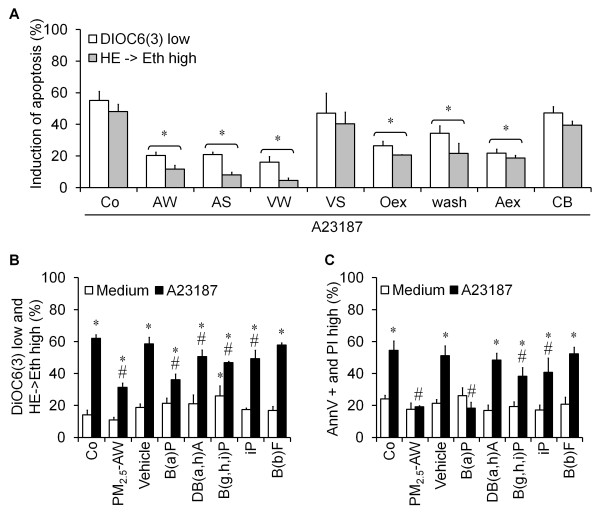
**Role of the different components of PM_2.5 _in the antiapoptotic effect**. (A) 16HBE cells were treated 4 h with different batches of PM_2.5 _10 μg/cm^2 ^(AW, AS, VW and VS), the equivalent concentration of organic extracts (Oex, 4.27 mg/ml), aqueous extracts (Aex), washed particles (wash, 10 μg/cm^2^) of PM_2.5_-AW and carbon black (CB, 10 μg/cm^2^) before a 20 h exposure to A23187 (3 μM). Apoptosis was assessed by flow cytometry and expressed as induction of apoptosis. Results are mean ± SD (n = 3). * *p *< 0.001 compared with A23187 alone. (B **and C**) Effect of heavy PAH on A23187-induced apoptosis. Treatments with PM_2.5_-AW (10 μg/cm^2^), the vehicle (Cylohexane, 1%), Benzo[a]pyrene (B(a)P, 270 nM), Dibenzo[a,h]anthracene (D,B(a,h)A, 35 nM), Benzo[g,h,i]perylene (B(g,h,i)P, 443 nM), Indeno[1,2,3-cd]pyrene (iP, 217 nM) and Benzo[b]fluoranthrene (B(b)F, 333 nM), were performed 4 h prior to induction of apoptosis by A23187 (3 μM). Results are mean ± SD (n = 4). * vs. control, *p *< 0.001; # vs. A23187, *p *< 0.05.

In order to identify the components of PM_2.5 _involved in the process of the antiapoptotic effect described herein, we compared the capacity of the four different batches of Parisian PM_2.5 _to reduce apoptosis mediated by A23187. Surprisingly, solely PM_2.5_-VS were unable to reduce apoptosis (Figure 6A) suggesting that the antiapoptotic effect of PM_2.5 _might be associated with some compounds which are less present in PM_2.5_-VS batch than in the others. In opposite, the lack of antiapoptotic effect might also be attributed to components more absorbed in PM_2.5_-VS than the others. Indeed, chemical analysis of all batches showed that PM_2.5_-VS contain more metals and less organic compounds (especially heavy PAH) than PM_2.5_-AW, AS and VW batches [2]. Thus, we tested PM_2.5_-AW organic extracts (Oex) and washed particles devoid of water-soluble components, PM_2.5_-AW aqueous extracts (Aex) and 95nm-carbon black particles (CB). Figure 6A shows that aqueous and organic extracts and, in a less extent washed particles, can mimic the antiapoptotic activity of whole PM_2.5_. In contrast, CB particles were unable to protect from apoptosis triggered by A23187. This suggests that water-soluble as well as organic compounds might be responsible for the antiapoptotic effect. To confirm this, we performed experiments with different heavy PAH (with at least 4 rings), such as Benzo[a]pyrene (B(a)P), Dibenzo[a,h]anthracene (DB(a,h)A), Benzo[g,h,i]perylene (B(g,h,i)P), Indeo[1,2,3-cd]pyrene (iP) and Benzo[b]fluoranthrene (B(b)F). Except for B(b)F, all the other PAH reduced the amount of apoptosis induced by A23187, similar to that of PM_2.5 _pretreatment and B(a)P seems to be the most efficient (Figure 6B and 6C). Note that the particle-coupled PAH are bioavailable in our system since CYP1A1 mRNA and its enzymatic activity were increased (data not shown). Moreover, when different light PAH (3-rings) found on particles were tested, the antiapoptotic effect was not found (Additional file 1 Figure S1). We also took into consideration the effect of biological compounds adsorbed onto particles, such as endotoxines, by using a specific bacteria LPS neutralizing protein rENP. This did not diminish the protector effect of PM_2.5 _from apoptosis induced by A23187 and STS (Additional file 1 Figure S2) indicating that endotoxins are not involved in the process. Altogether, our data strongly suggest that water-soluble and heavy PAH components contribute to the antiapoptotic effect of Parisian PM_2.5 _observed in human bronchial epithelial cells.

### The antiapoptotic mechanism is mediated by the aryl hydrocarbon receptor

**Figure 7:**
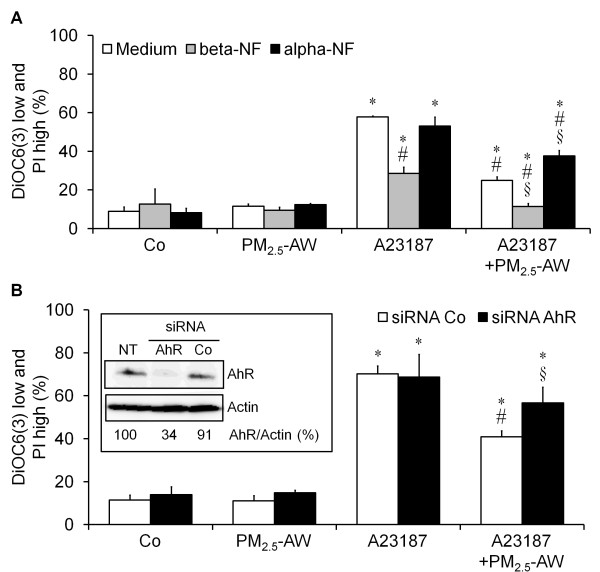
**AhR pathway is involved in the antiapoptotic effect of Parisian PM_2.5_**. (A) 16HBE epithelial cells were preincubated or not in the presence of the agonist beta-naphtoflavone (beta-NF, 20 μg/ml) or the antagonist alpha-naphtoflavone (alpha-NF, 10 μg/ml) one hour before the usual PM_2.5_-AW pretreatment and/or apoptosis inducer A23187 (3 μM). Apoptosis was measured by flow cytometry as above. Results are mean ± SD (n = 3). *p *< 0.001, * vs. Control, # vs. A23187, § vs. A23187 + PM_2.5_. (B) Effect of AhR silencing. (Upper panel) 16HBE cells were incubated during 48 h with AhR siRNA (AhR, 10 nM), a control siRNA (Co, 10 nM) or non-transfected (NT), then total cell extract (80 μg) were loaded onto gel and subjected to immunoblotting with anti-AhR and anti-Actin antibodies. Quantifications were performed as respect to Actin and illustrated as AhR/Actin ratio. (Lower Graph) 16HBE cells were incubated with siRNA for 48 h as above and then 4 h pretreated to PM_2.5_-AW before apoptosis induction with A23187 for 20 h supplementary. Results of flow cytometry (DiOC(6)3 low and IP high) are from transfected cells (Alexa Fluor 647 positive population) which were around 80% for all conditions, illustrated as mean ± SD (n = 4). *p *< 0.001, * vs non-treated siRNA Co; *p *< 0.001, # vs. A23187 siRNA Co; *p *< 0.005, § vs. A23187 + PM_2.5 _siRNA Co.

To delineate the molecular mechanism of the antiapoptotic effect of PM_2.5 _efficient at the mitochondrial checkpoint, we focused on the aryl hydrocarbon receptor (AhR) activated after cell exposure to organic compounds such as PAH. Indeed, AhR is a ligand-induced transcription factor which relocates to the nucleus and induces the expression of numerous target genes. Thus, we investigated the possible implication of AhR in our process. To test this we first either activated or inhibited AhR, using an agonist (beta-naphtoflavone, beta-NF) or an antagonist (alpha-naphtoflavone, alpha-NF). Figure 7A shows that beta-NF (20 μg/ml) used prior to A23178 significantly reduced the amount of apoptotic cells (31% reduction of DiOC6(3) low), and further improved the protection conferred by PM_2.5 _exposure (54% vs. 37% reduction of DioC6(3) low). Conversely, pretreatment with alpha-NF significantly reduced the protection provided by PM_2.5 _exposure (25% vs. 37% reduction of DiOC6(3) low), although it did not noticeably modify the apoptotic effect of A23187. These findings are consistent with the involvement of AhR in the antiapoptotic effect of PM_2.5 _exposure.

Finally, we tested the effect of AhR silencing in the antiapoptotic effect observed after PM_2.5_-exposure. For this, we used validated fluorescent-siRNA (Alexa Fluor 647) in order to select the fluorescent positive cells by flow cytometry. After siRNA optimization (80% transfected cells with 10 nM siRNA after 48 h, data not shown) and validation of AhR silencing by western blot (Figure 7B upper panel), DiOC(6)3 and PI assays were performed by flow cytometry on cells exposed or not to PM_2.5 _and/or A23187 for 24 h as before. Figure 7B (lower graph) shows that AhR silencing significantly reduced the protection triggered by PM_2.5 _(18% vs. 27% reduction of DiOC(6)3 low) alike the antagonist (alpha-NF) did. Interestingly, both the AhR silencing and AhR antagonist partially reduced the PM_2.5_-protective effect with almost the same extent (10%). The increase in alpha-NF concentration (20 μg/ml, data not shown) or siRNA-AhR amount (25 nM, Additional file 1 Figure S3) did not completely abolish the protection suggesting that another pathway might be involved. Taken together, these results suggest that AhR partially contributes to the antiapoptotic effect of PM_2.5 _exposure.

## Discussion

To our knowledge, this article is the first study presenting evidence that low concentrations of PM which are not cytotoxic, have an antiapoptotic effect on human bronchial epithelial cells. We report here the cellular effects of PM_2.5 _from two sites in Paris, sampled in winter and in summer. In order to remove the risk of cell type-specific events, our study was done in parallel on different human bronchial cell lines as well as on primary cells. We show that the four batches of PM_2.5 _are not cytotoxic on human bronchial cells, at a range of concentration from 1 to 50 μg/cm^2^. This is supported by data from flow cytometry, with the measurement of the main apoptotic hallmarks, as well as from electron microscopy data. Our results were obtained with a low concentration of PM_2.5 _unlike previous publications performed with higher doses (e.g. 100 μg/cm^2^, [18]). Indeed, the standard dose used here (10 μg/cm^2^) is a concentration which could mimic a five day exposure of PM_2.5 _in the tracheobronchial region, considering that PM_2.5 _mass deposition is 2.3 μg/cm^2^/24 h [11]. Our results are in agreement with a previous publication where BEAS-2B human bronchial cells were not susceptible to diesel exhaust particles-induced apoptosis [27] and here, we provided supplementary evidences of a non-toxicological activity of PM_2.5 _in NHBE primary culture. Moreover, in our studies and those of Sanchez-Perez *et al. *[28], the lack of induced-apoptosis triggered by PM at 10 μg/cm^2 ^suggests that a "sub-lethal" concentration could have different impacts on cell fate than at high concentrations.

The originality of this work is that PM_2.5 _exposure confers a specific decrease in apoptosis induced by A23187, staurosporine and oligomycin as demonstrated in immortalized (16HBE), cancerous (NCI-H292, BEAS-2B) as well as primary normal bronchial epithelial cells (NHBE). In order to characterize the molecular mechanism of the antiapoptotic activity of PM_2.5 _exposure, first we demonstrated that the reduction of apoptosis is observed prior to proinflammatory cytokines secretion which led us to rule out the involvement of the classical EGFR signaling pathway as well as the proinflammatory cytokines secretion by bronchial epithelial cells. However, PM_2.5_-antiapoptotic effect in addition to the well-documented inflammatory response might explain the maintenance of a prolonged inflammation state *in vivo *induced after pollution exposure and might delay repair processes of injured tissues [29].

To further delineate the mechanism of the antiapoptotic activity, a strategy would be to identify the cellular targets which are in common between staurosporine, A23187 and oligomycin. On one hand, staurosporine and A23187 are known to regulate cellular calcium signaling pathways inducing an endoplasmic reticulum stress which leads to cytoplasmic calcium uptake [24], mitochondrial Ca^2+ ^overload [30] and finally ΔΨm drop. Thus, PM_2.5 _exposure might counteract the Ca^2+ ^uptake induced by these apoptotic inducers. However, this hypothesis is in discrepancy with the fact that the antiapoptotic effects of PM_2.5 _were not observed when using ionomycin, which is a well-known calcium ionophore, like A23187. Indeed, A23187 and ionomycin, which are both monocarboxylic ionophores, promote a selective increase of cytosolic Ca^2+ ^[31]. But on the contrary to A23187 [24], a recent study showed that ionomycin did not allow the mitochondrial calcium overload in epimastigote cells of *Trypanosoma cruzi *[32]. The measurement of cytosolic and mitochondrial calcium uptakes in response to A23187 and ionomycin might allow us to understand why A23187-induced apoptosis is sensitive to PM while ionomycin is not. Moreover, caspases are the main effectors of apoptosis, but A23187, staurosporine and ionomycin can also activate Ca^2+^-specific proteases, such as calpains [33,34]. Indeed, our preliminary studies showed that calpains are activated after A23187 treatment of 16HBE and NCI-H292 cells (data not shown). As described for oligomycin, A23187, but not ionomycin, is a specific inhibitor of mitochondrial ATP synthase also known to catalyze the direct exchange of Ca^2+^/2H^+ ^in liver mitochondria [35] and to disrupt the mitochondrial transmembrane potential [36]. All these data suggest that ionomycin and A23187 might trigger the apoptotic process by slightly different mechanisms especially at the mitochondrial level. Thus, we hypothesize that PM_2.5 _could directly reduce apoptosis at the mitochondrial step by maintaining ΔΨm, or *via *the upregulation of antiapoptotic proteins such as Bcl-2 known to protect from A23187-induced apoptosis [24,37].

Humans are exposed to a mixture of compounds including organic and inorganic components adsorbed on PM. Evidences suggest that organic compounds such as the polycyclic aromatic hydrocarbons (PAH) can mimic the pro-oxidant [3] and apoptotic effect of PM [38]. Here, we investigated the role of different organic compounds (PAH, Oex), particles devoid of hydrosoluble components, (wash, CB) and aqueous extracts (Aex) of PM_2.5 _with respect to cell death. We found that the organic extracts and several heavy PAH, B(a)P in particular, could reproduce the antiapoptotic activity. Moreover, the water-soluble fraction also contributes to the reduction of apoptosis while carbon black, light PAH and endotoxins have no effect. In our study, B(a)P is the compound that protects the most efficiently from apoptosis induced by A23187. This points out a possible link between PM_2.5_-exposure and the antiapoptotic effect observed herein, as also suggested by Hung *et al. *[39]. The harmful health impacts of PAH are well-known, like the promotion of cancers. B(a)P-diones, which are photomodified by the sunlight, were also found in air particulate matter. In agreement with our results, a recent work demonstrated that sunlight-exposed B(a)P inhibits apoptosis induced by cell detach**ment **[40]. B(a)P is metabolized by cells, transformed into a reactive intermediate (anti-7,8-dihydrodiol-9,10-epoxy-benzo[a]pyrene, BPDE) that causes DNA damage and mutations in tumor suppressor genes, such as p53 [41]. This toxic metabolite BPDE is also capable to suppress apoptosis of mammary epithelial cells [42].

The main cellular target of PAH adsorbed on PM is the aryl hydrocarbon receptor (AhR), thus we addressed the question of AhR involvement in the antiapoptotic effect after PM_2.5 _exposure. We showed here that the activation of AhR by the agonist beta-naphtoflavone improves the antiapoptotic effect. On the contrary, the inhibition of AhR (using **a **specific inhibitor or RNA silencing) diminished the antiapoptotic effect suggesting that AhR is involved in this process. An additional argument is brought by the absence of antiapoptotic activity when we tested light PAH, which were previously shown to poorly promote AhR activation [43]. AhR is a cytoplasmic ligand-dependent transcription factor which translocates to the nucleus in order to bind specific Xenobiotic Responsive Elements in the promoter of its target genes, leading to the activation of phase I and II metabolizing enzymes and thus contributing to detoxification [44]. But in the absence of ligand, many data suggest other roles than detoxification [45] and recent evidences suggest that AhR inactivation could modify the expression of numerous genes, including those involved in cell cycle regulation [46]. In accordance with our results, other publications suggest an antiapoptotic activity of AhR by a direct interaction with E2F1 leading to the reduction of E2F1-mediated pro-apoptotic genes expression [47]. This is consistent with the idea that the AhR might modulate cell death at the mitochondrial checkpoint, for instance by upregulating the expression of antiapoptotic *bcl-2*, *bcl-xL, mcl-1 or agr2 *genes [48,49] or by repressing the pro-apoptotic *apaf-1 *[47]. Moreover, AhR might indirectly regulate apoptosis through the MMP step by increasing the expression of the anti-apototic protein VDAC2 [50] which is known to participate to the permeability transition pore (PTP) and which also bind to and inhibit the apoptotic protein Bak [51]. In the light of our observations, it will be interesting to find out the genes encoding mitochondrial regulators which are modulated by AhR and involved in the protection observed after PM_2.5_-exposure or B(a)P treatment. It is also important to point out that both A23187 and STS could induce apoptosis *via *a Ca^2+^-dependent pathway through mitochondrial PTP opening and that VDAC plays a crucial role in the transport of Ca^2+ ^into this organelle [52].

## Conclusion

**Figure 8:**
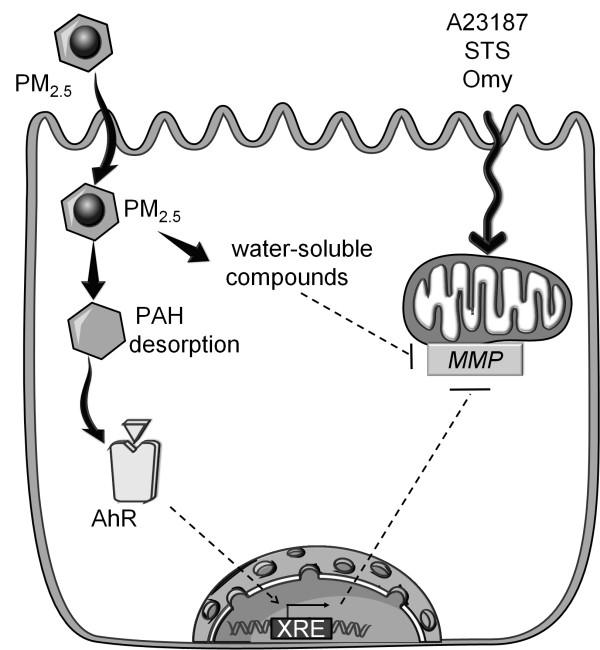
**Hypothetic model for the mechanism of the antiapoptotic effect of PM_2.5_**. Exposure of bronchial epithelial cells to PM_2.5_, leads to particles endocytosis and PAH desorption. Then, intracellular PAH can target and activate the aryl hydrocarbon receptor (AhR). It is noteworthy, that AhR translocates to the nucleus to bind to specific xenobiotic responsive elements (XRE) in the promoter of its target genes; some of them might be mitochondrial MMP regulators (dotted arrow), thus might protect the cell from apoptosis induced by A23187, staurosporin (STS) or oligomycin (Omy). Moreover, the water-soluble compounds of PM_2.5 _also have antiapoptotic activity, but the pathway involved is still under investigation (dotted arrow). Illustration carried out thanks to Servier Medical Art.

In summary, Parisian PM_2.5 _are not cytotoxic in four cellular models of bronchial epithelial cells. However, PM_2.5 _exposure rapidly triggers an antiapoptotic effect at the mitochondrial level, which seems to be linked to the water-soluble and some PAH components adsorbed on particles. Finally, the AhR pathway partially contributes to the antiapoptotic effect of fine particles. Altogether, our results allow us to propose the hypothetic model in which desorbed PAH may activate the AhR leading to the regulation of genes involved in the mitochondrial checkpoint of apoptosis (Figure 8). In parallel, the water-soluble fraction seems to have similar effect on mitochondria by regulating unknown pathways. Our results are the first evidence of a missing link in the connection between adverse health effects of fine particles and exacerbation of cancerous pathologies, via the cell death impediment in their presence. Furthermore, the antiapoptotic effect of PM_2.5 _associated with the well-documented inflammatory response might also explain the maintenance of a prolonged inflammation state *in vivo *induced after pollution exposure.

## Materials and methods

### Particles collection

Urban atmospheric PM_2.5 _were collected during winter or summer 2003 at two locations in Paris: an urban background station at Vitry-sur-Seine (VW: Vitry winter and VS: Vitry summer), a suburb of Paris; and a curbside station at Porte d'Auteuil bordering the highway ring road of Paris (AW: Auteuil winter and AS: Auteuil summer). Particles were recovered on 150 mm diameter nitrocellulose filters (HAWP, Millipore, Saint-Quentin-en-Yvelines, France) with a high volume sampler machine (DA-80 Digitel, Switzerland, flowrate: 30 m^3^/h, [6]). Their PAH and metal content have been previously described [2]. PM_2.5_-AW organic extracts (Oex) were obtained after extraction by dichloromethane, then dried and redissolved in dimethyl sulfoxide (DMSO, Sigma). Oex were used at the concentration found on particles according to the soluble organic fraction (SOF) determined for PM_2.5_-AW particle sample (10%). The aqueous extract of PM_2.5_-AW (Aex) containing hydrosoluble components was obtained after the washing of the particle suspension and two centrifugations at 10,000 g, followed by filtration of the supernatant through a 0.22 μm Durapore^® ^filter (polyvinylidine difluoride) (Millex^® ^GV, Carrigtohill, Cork, Ireland). Cells were exposed to a volume of aqueous extract equivalent to the volume of particle suspension used. Particles collected after the two centrifugations constitute the washed PM_2.5_-AW (wash) devoid of hydrosoluble components. Carbon black particles (CB, Fr101, 95 nm, 20 m^2^/g) were purchased from Degussa (Frankfurt, Germany). All particles were stored in DMEM medium and used at standard dose 10 μg/cm^2 ^(40 μg/ml). For treatment, after thawing, particles were sonicated three times for 20 s at 70W (Vibracell, Bioblock Scientific, Illkrich, France) and added directly onto the cells. Purified PAH, B(a)P, DB(a,h)A, B(g,h,i)P, iP, B(b)F, PA, FA and vehicle cyclohexane were purchased from Sigma.

### Cell culture conditions

Human bronchial epithelial cells 16HBE14o- kindly provided by Dr. D.C. Gruenert (Colchester, VT, USA [53], were cultured in DMEM/F12 medium (Invitrogen) supplemented with 2 mM GlutaMAX™-I, 100 U/ml penicillin, 100 μg/ml streptomycin, 1.25 μg/ml fungizone and 2% UltroserG (UG, BioSepra, France). Cells were grown to subconfluence on bovine collagen (Purecol Natucan, 0.5 mg/cm^2^) and human fibronectin coating (Sigma, 4 μg/cm^2^). Prior to particle treatment, UG was removed. BEAS-2B human bronchial epithelial cells (provided by Dr. J. Boczkowski, Faculté de Médecine Xavier Bichat, Université Paris 7, France) were cultured in LHC-9 medium containing retinoic acid (33 nM). The human lung mucoepidermoid carcinoma cells (NCI-H292) were purchased from the American Type Culture Collection (Rockville, MD) and cultured in RPMI-1640 medium (Invitrogen) supplemented with 1% GlutaMAX™-I and 10% fetal calf serum. Primary cultures of normal human bronchial epithelial (NHBE) cells were obtained from Lonza and cultured for in Clonetics^® ^BEGM medium (Cambrex, Walkersville, MD, USA) supplemented with EGF 25 ng/ml. During treatment NHBE cells were grown in DMEM/F12 without growth factors.

### Chemicals and apoptosis measurement

Cells were exposed 4 h to PM_2.5 _(1-50 μg/cm^2^) prior to addition of apoptotic inducers for additional 20 hours: rotenone (Rot, 5 μM), antimycin (AMA, 25 μg/ml), oligomycine (Omy, 5 μM), ionomycin (Iono, 0.5 μM), A23187 (3 μM), staurosporine (STS, 1 μM) and hydrogen peroxide (H_2_O_2_, 500 μM). All drugs were purchased from Sigma. Apoptotic parameters were quantified by flow cytometry performed on CyAn ADP LX (Dako Cytomentation funded by the Ligue Nationale contre le Cancer R03/75-79) using 3, 3 dihexyloxacarbocyanine iodide (DiOC_6_(3), 2 nM) for ΔΨm quantification, 10 μg/ml propidium iodide (PI) for determination of plasma membrane permeabilization, 2 μM hydroethidine (HE, Molecular Probes) for superoxide anion generation, and Annexin V conjugated with fluorescein isothiocyanate (FITC, Sigma) for the assessment of phosphatidylserine (PS) exposure [54]. Percentage of induction of apoptosis is calculated according to the following formula: % = 100 × (% of apoptotic treated cells - % of apoptotic control cells)/(100 - % of apoptotic control cells). Recombinant EGF (rEGF) and EGFR inhibitor (AG1478) are from Sigma. The specific AhR antagonist alpha-naphthoflavone (alpha-NF, 10 μg/ml, Sigma) or agonist beta-naphthoflavone (beta-NF, 20 μg/ml, Sigma) were used for 1 h prior to PM_2.5 _exposure and/or apoptosis induction.

### Electron Microscopy

Cells were fixed 1 h by immersion at 4°C in 2.5% glutaraldehyde and 1% tannic acid in 0.1 M sodium cacodylate buffer, washed, postfixed in 2% osmium tetroxide deshydrated before embedding in Epon. Electron microscopy was performed with a transmission electron microscope (model Philips TECNAI 12), at 80 kV on ultrathin sections (60 nm).

### Amphiregulin and GM-CSF secretion

Subconfluent 16HBE cells were exposed to PM_2.5_-AW for 4 h or 24 h and supernatants were recovered, centrifuged at 15,000 × g for 15 min at 4°C to pellet particles, and then frozen at -80°C until further analysis. The concentrations of Amphiregulin (AR) and GM-CSF released were evaluated with an enzyme-linked immunosorbent assay kit (ELISA, R&D Systems Europe; Abingdon, UK) according to the manufacturer's recommendations.

### AhR gene silencing

16HBE cells were simultaneously seeded at 2 × 10^4 ^cells/cm^2 ^either in T25 dishes (for Western Blot) or in a P24 well plate (for flow cytometry) and incubated under normal cell culture conditions overnight. Then, 10 nM of AhR siRNA (Hs_AHR_6 AlexaFluor-647, Ref SI03043971, Qiagen) or control non-silencing siRNA (AllStars Neg AlexaFluor-647, Ref 1027287, Qiagen) and HiPerFect Transfection Reagent (Qiagen) were mixed separately in medium and the formed-complexes were then added drop-wise onto the cells, according to the manufacturer's recommendations. At 48 h after transfection, the cells were subjected to our usual protocol: 4 h PM_2.5 _pretreatement and/or A23187 (3 μM) for additional 20 h.

### Western Blots

Western Blots were performed according to the method previously described [55] and the primary antibodies used were: mouse-monoclonal anti-AhR (WH0000196M2, Sigma) and anti-Actin (A5441, Sigma). The secondary antibodies (peroxidase-conjugated) were anti-mouse immunoglobulin (A9044, Sigma). Immunoreactive bands were detected by chemiluminescence using a Chemiluminescent Sensitive HRP Substrate (BioFX Laboratories) using a FujiFilm LAS 4000 camera system.

### Statistical analysis

All results are presented as the mean +/- standard deviation of three independent experiments. Data were analyzed using one-way ANOVA analysis of variance. The Dunnett's test was performed for all multiple comparisons versus control group. Moreover, the Student-Newman-Keuls test was used for all pairwise comparisons of mean responses among the different treatment groups (SigmaStat). Differences between groups were considered significant if the *p *value was less than 0.05.

## Abbreviations

A23187: calcium ionophore (calcimycin); Aex: aqueous extracts; AhR: aryl hydrocarbon receptor; alpha-NF: alpha-naphtoflavone; AMA: antimicyn A; AR: Amphiregulin; B(a)P: benzo[a]pyrene; B(b)F: benzo[b]fluoranthrene; B(g,h,i)P: benzo[g,h,i]perylene; beta-NF: beta-naphtoflavone; COPD: chronic obstructive pulmonary diseases; DB(a,h)A: dibenzo[a,h]anthracene; DEP: Diesel exhaust particles; DiOC_6_(3): 3, 3 dihexyloxacarbocyanine iodide; EGF: epidermal growth factor; FA: fluoranthene; FITC: fluorescein isothiocyanate; GM-CSF: granulocyte monocyte colony-stimulating factor; H_2_O_2_: hydrogen peroxide; HE: hydroethidine; Iono: ionomycin; iP: indeo[1,2,3-cd]pyrene; LPS: lipopolysaccharide; MMP: mitochondrial membrane permeabilization; Oex: organic extracts; Omy: oligomycin; PA: phenanthren; PAH: polycyclic aromatic hydrocarbons; PI: propidium iodide; PM: particulate matter; PM_2.5_-AS: Auteuil summer; PM_2.5_-AW: Auteuil winter; PM_2.5_-VS: Vitry summer; PM_2.5_-VW: Vitry winter; PS: phosphatidylserine; ROS: reactive oxygen species; Rot: rotenone; siRNA: small interfering RNA; STS: staurosporine; wash: washed particles; ΔΨm: mitochondrial transmembrane potential.

## Competing interests

The authors declare that they have no competing interests.

## Authors' contributions

IF and KA designed the research, analyzed the data and drafted the paper. IF, MCB, CB, ML, NB and KA performed the experiments. AB and FM contributed to the interpretation of the data and helped improving the manuscript. All authors read and approved the final manuscript.

## Funding information

This work was supported by Agence Nationale de la Recherche [0599-5 SET 024-01], Centre National de la Recherche Scientifique (CNRS), Université Paris Diderot-Paris 7 (Bourse de Master, Melanie Leroux), Région Ile de France (Allocation post-doctorale, Ioana Ferecatu [F-08-1261/R]), ADEME-Primequal [0462C0056], CAMPLP (Caisse d'Assurance Maladie des Professions Liberales de Province, Paris, France), Renault (DIMAT, for the supply and chemical analysis of PM_2.5 _and PM organic extracts) and Legs Poix.

## Supplementary Material

Additional file 1 **Figure S1: The antiapoptotic effect of PM2.5 is not related to light three-rings PAH**. **Figure S2**: The antiapoptotic effect of PM_2.5 _is not related to the adsorbed endotoxins. **Figure S3**: Higher amounts of AhR siRNA do not completely abolish the antiapoptotic effect of PM_2.5 _exposure. Click here for file
