# Supplementary material for: Polycyclic aromatic hydrocarbon components contribute to the mitochondria-antiapoptotic effect of fine particulate matter on human bronchial epithelial cells via the aryl hydrocarbon receptor
Source: Part Fibre Toxicol. 2010 Jul 21;7:18. doi: 10.1186/1743-8977-7-18 (PMC2914693; doi:10.1186/1743-8977-7-18)
Supplement: Additional file 1 — Figure S1: The antiapoptotic effect of PM2.5 is not related to light three-rings PAH. Figure S2: The antiapoptotic effect of PM2.5 is not related to the adsorbed endotoxins. Figure S3: Higher amounts of AhR siRNA do not completely abolish the antiapoptotic effect of PM2.5 exposure. [file 1743-8977-7-18-S1.PDF]

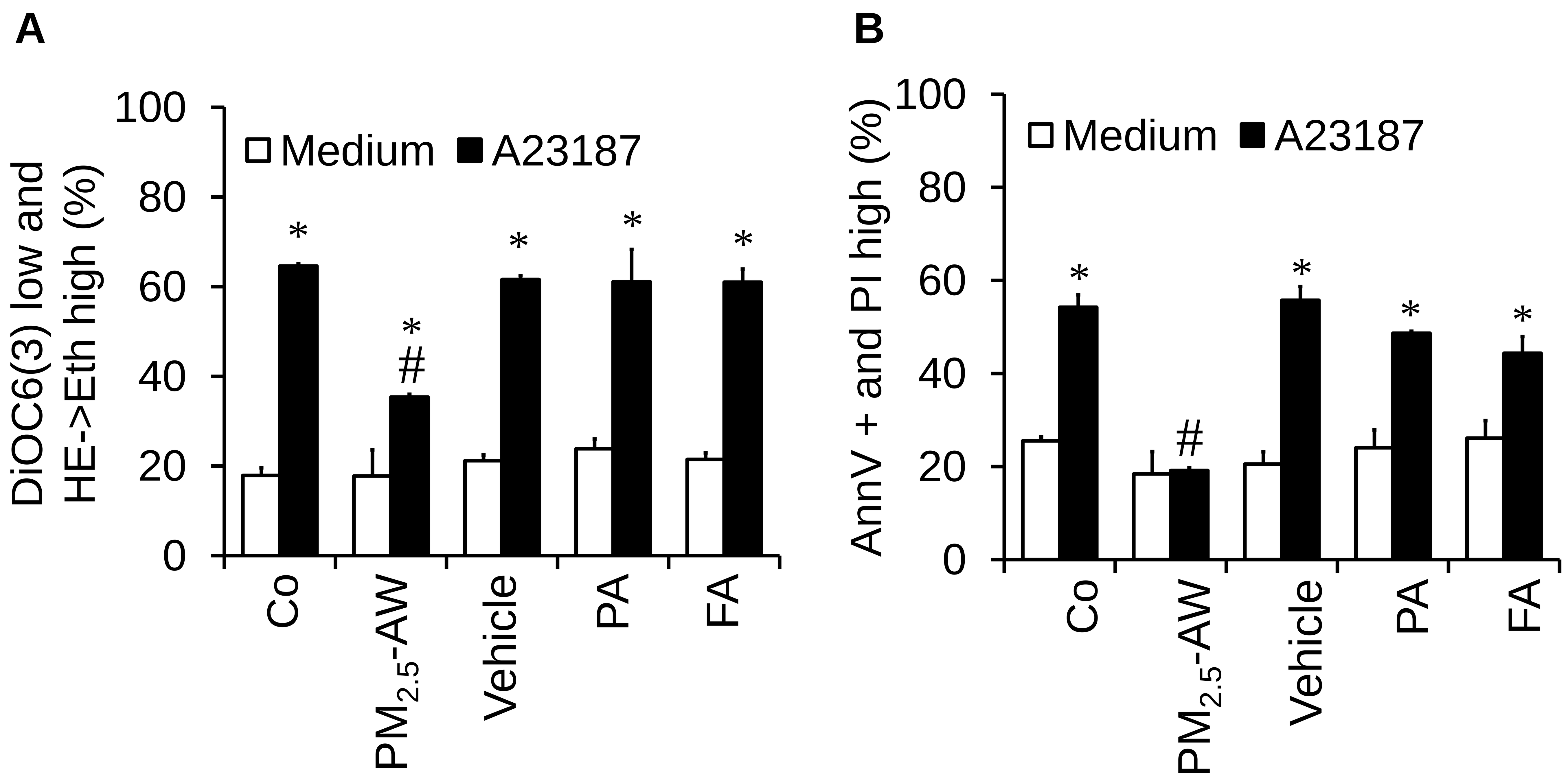

Figure S1: The antiapoptotic effect of $PM_{2.5}$ is not related to light three-rings PAH. Epithelial 16HBE cells were pretreated with $PM_{2.5}$-AW (10 µg/cm²), the vehicle (Cylohexane, 1%), Phenanthrene (PA, 124 nM), and Fluoranthene (FA, 268 nM) 4 h prior to apoptotic induction by A23187 (3 µM) and before flow cytometric analysis of cells presenting simultaneously DiOC6(3) low and HE high staining (A) or an Annexin V+ and PI high (B) . Results are mean ± SD (n=4). Significance was calculated with respect to control (*, p<0.001) or with respect to A23187 alone (#, p<0.001).

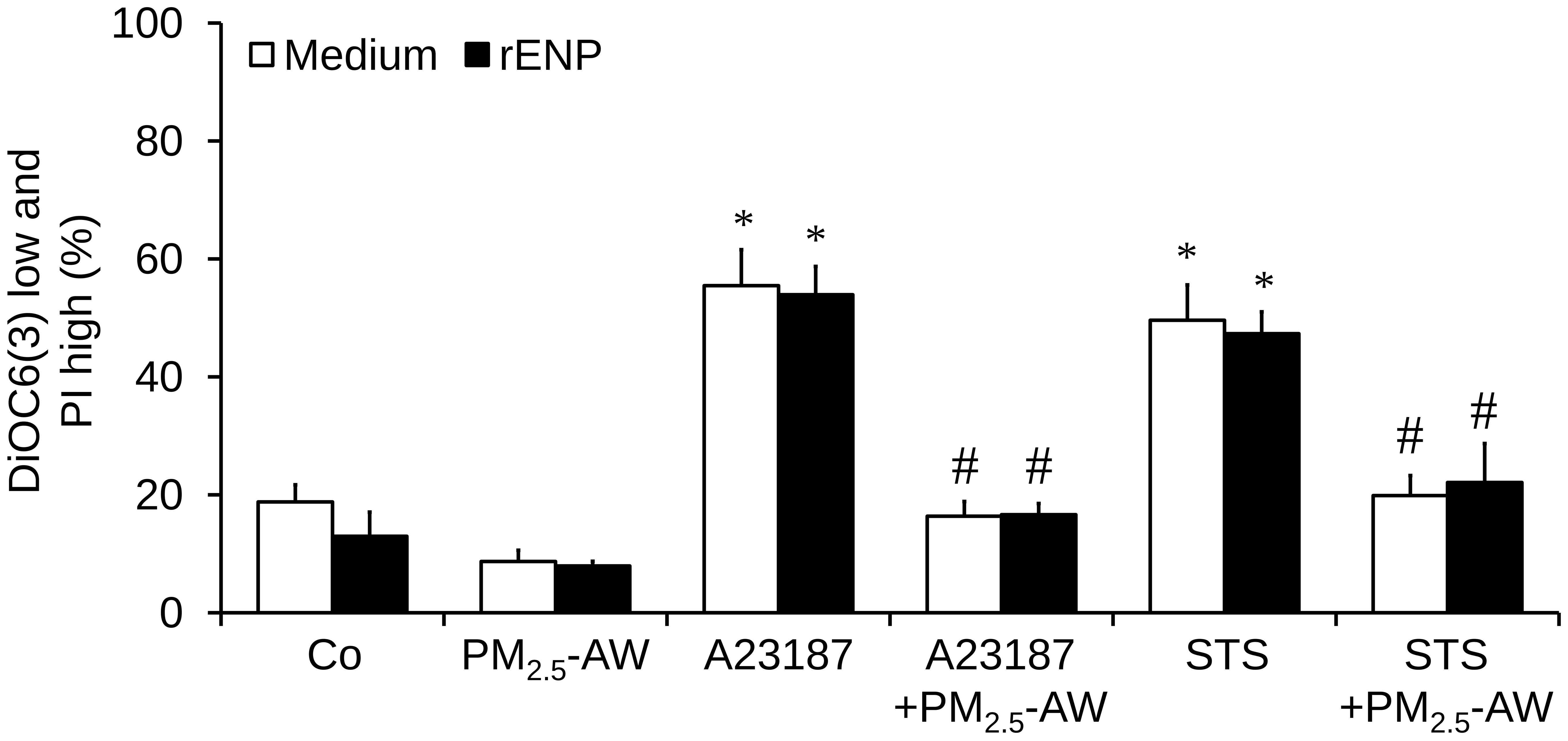

Figure S2: The antiapoptotic effect of $PM_{2.5}$ is not related to the adsorbed endotoxins. Epithelial 16HBE cells were pretreated 1 h with rENP (endotoxin neutralizing protein, 2 µg/µl) before the usual 4 h exposure to $PM_{2.5}$-AW (10 µg/cm²) and treated 20 h with A23187(3 µM) or staurosporine (STS, 1µM). Apoptosis was assessed by flow cytometry with the measurement of DiOC(6)3 low and PI high. Results are mean ± SD (n=3). Significance was calculated vs control (*, $p<0.001$), vs apoptosis inducer alone (#, $p<0.001$).

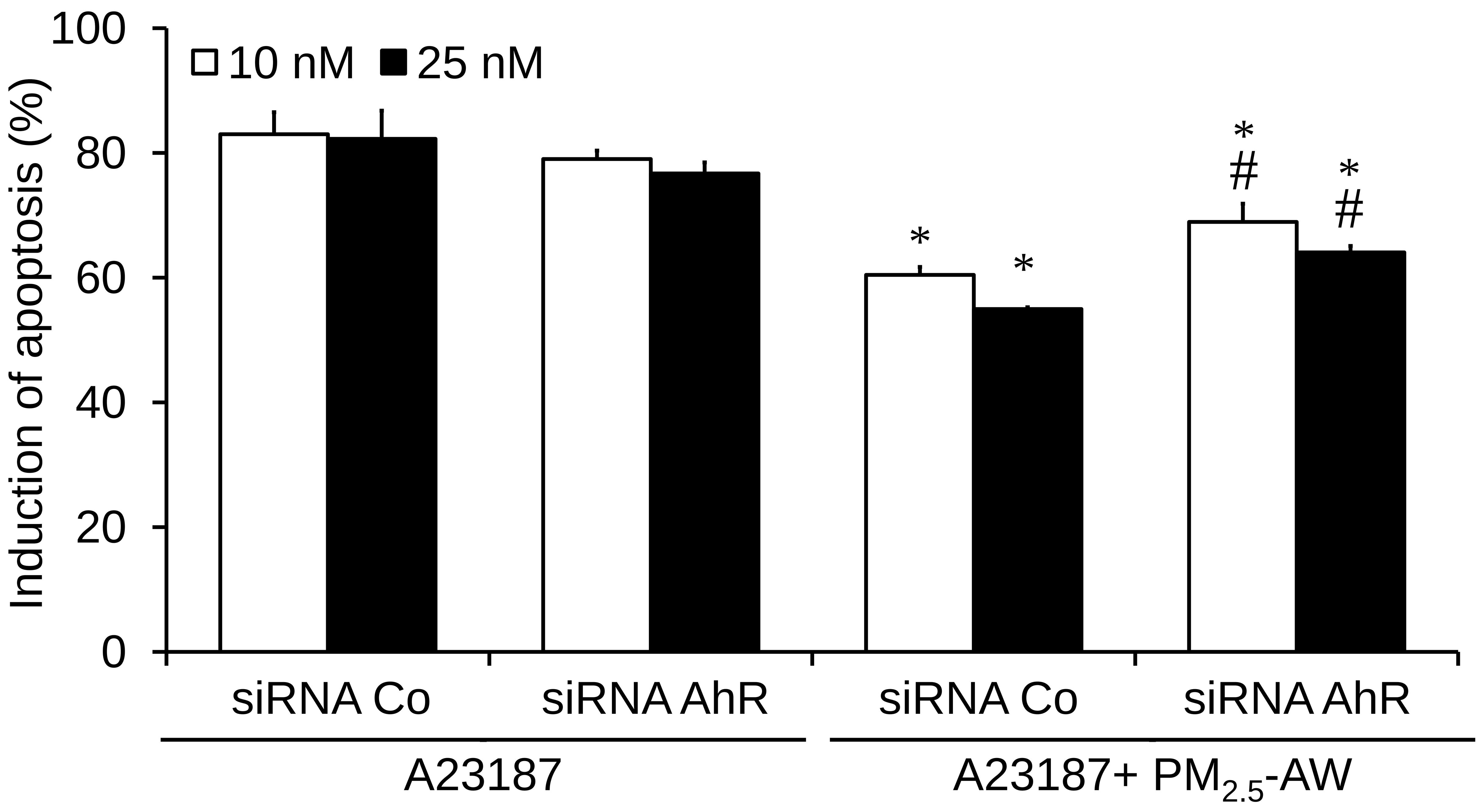

Figure S3: Higher amounts of siRNA AhR do not completely abolish the antiapoptotic effect of $PM_{2.5}$ exposure. 16HBE cells were incubated during 48 h with either 10 nM or with 25 nM of siRNA for control (siRNA Co) or AhR (siRNA AhR). Cells were then pretreated 4 h with $PM_{2.5}$-AW before induction of apoptosis with A23187 for additional 20 h. Results of flow cytometry (DiOC(6)3 low) are from Alexa Fluor 647 positive transfected cells, illustrated as mean ± SD (n=3). Significance was calculated with respect to A23187 siRNA Co (*, $p<0.001$), and with respect to siRNA Co for the A23187 + $PM_{2.5}$ condition (#, $p<0.01$).
